# Supplementary material for: Lateral hypothalamic proenkephalin neurons drive threat-induced overeating associated with a negative emotional state
Source: Nat Commun. 2023 Oct 28;14:6875. doi: 10.1038/s41467-023-42623-6 (PMC10613253; doi:10.1038/s41467-023-42623-6)
Supplement: Supplementary file 1 — Supplementary information [file 41467_2023_42623_MOESM1_ESM.pdf]

Supplemental information

Lateral hypothalamic proenkephalin neurons drive threat-induced overeating associated with a negative emotional state

In-Jee You, Yeeun Bae, Alec R. Beck, Sora Shin

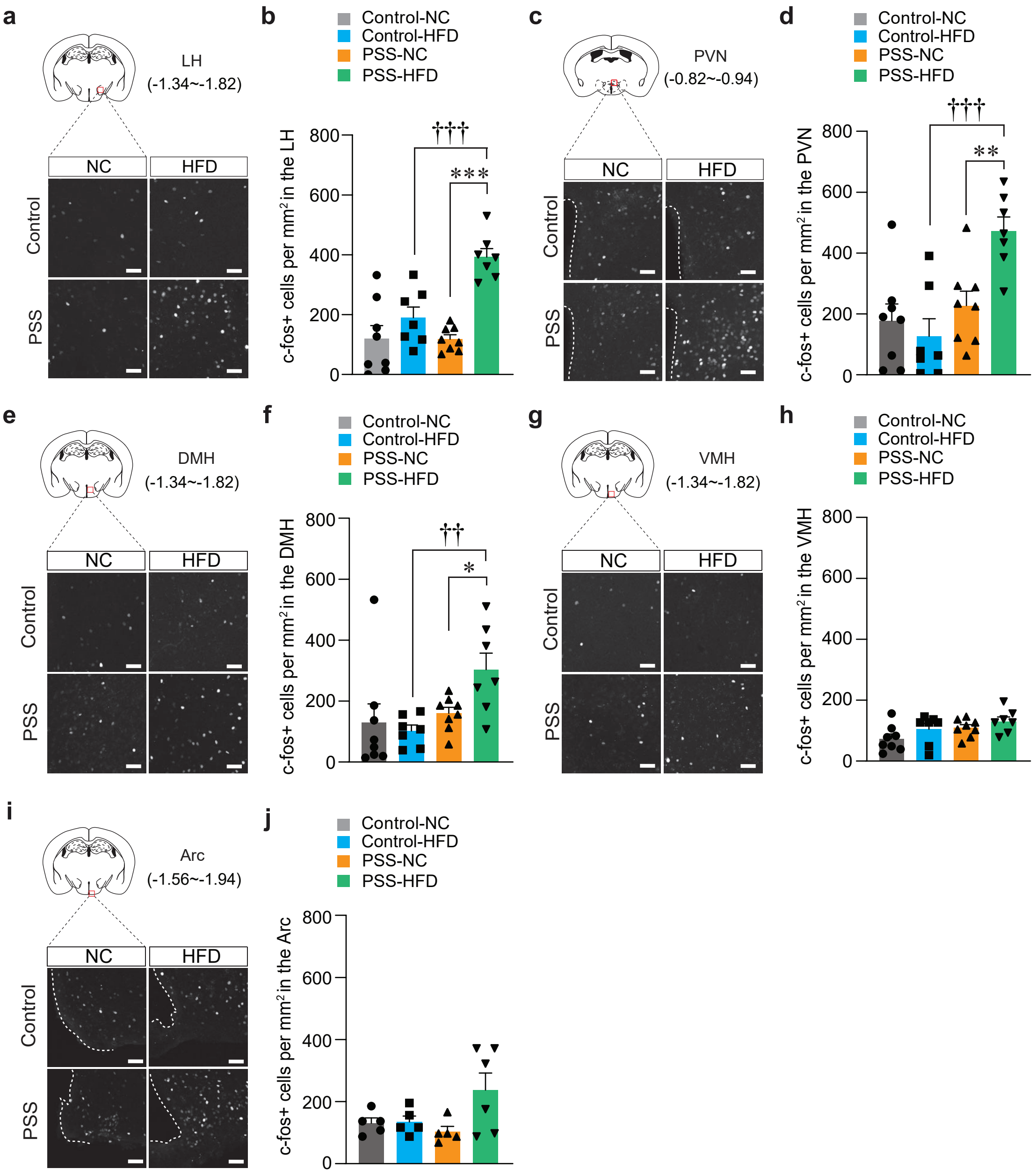

Supplementary Fig. 1

Supplementary Fig. 1 | HFD-induced c-fos expression in several hypothalamic subregions 24 h after PSS exposure.

(a-j) Coronal diagrams depicting the brain region analyzed for quantifying c-fos-positive cells (squared in red; top left). Representative images and quantifications of c-fos-positive cells in the LH (**a, b;** n = 8, 7, 8, and 7 mice per group), PVN (**c, d;** n = 8, 7, 8, and 7 mice per group), DMH (**e, f;** n = 8, 7, 8, and 7 mice per group), VMH (**g, h;** n = 8, 7, 8, and 7 mice per group) and Arc (**i, j;** n = 5, 5, 5, and 6 mice per group) of controls and PSS mice. Scale bars, 50 µm. Two-way ANOVA was followed by Fisher LSD post hoc test for multiple comparisons; In **b** ($F_{(1,26)}$= 10.006, p = 0.004), ***p < 0.001 compared with PSS mice exposed to NC, †††p < 0.001 compared with control mice exposed to HFD; In **d** ($F_{(1,26)}$= 8.044, p = 0.009), **p = 0.003 compared with PSS mice exposed to NC, †††p < 0.001 compared with control mice exposed to HFD; In **f** ($F_{(1,26)}$= 3.766, p = 0.063), *p = 0.029 compared with PSS mice exposed to NC, ††p = 0.004 compared with control mice exposed to HFD.

Data are presented as mean ± SEM.

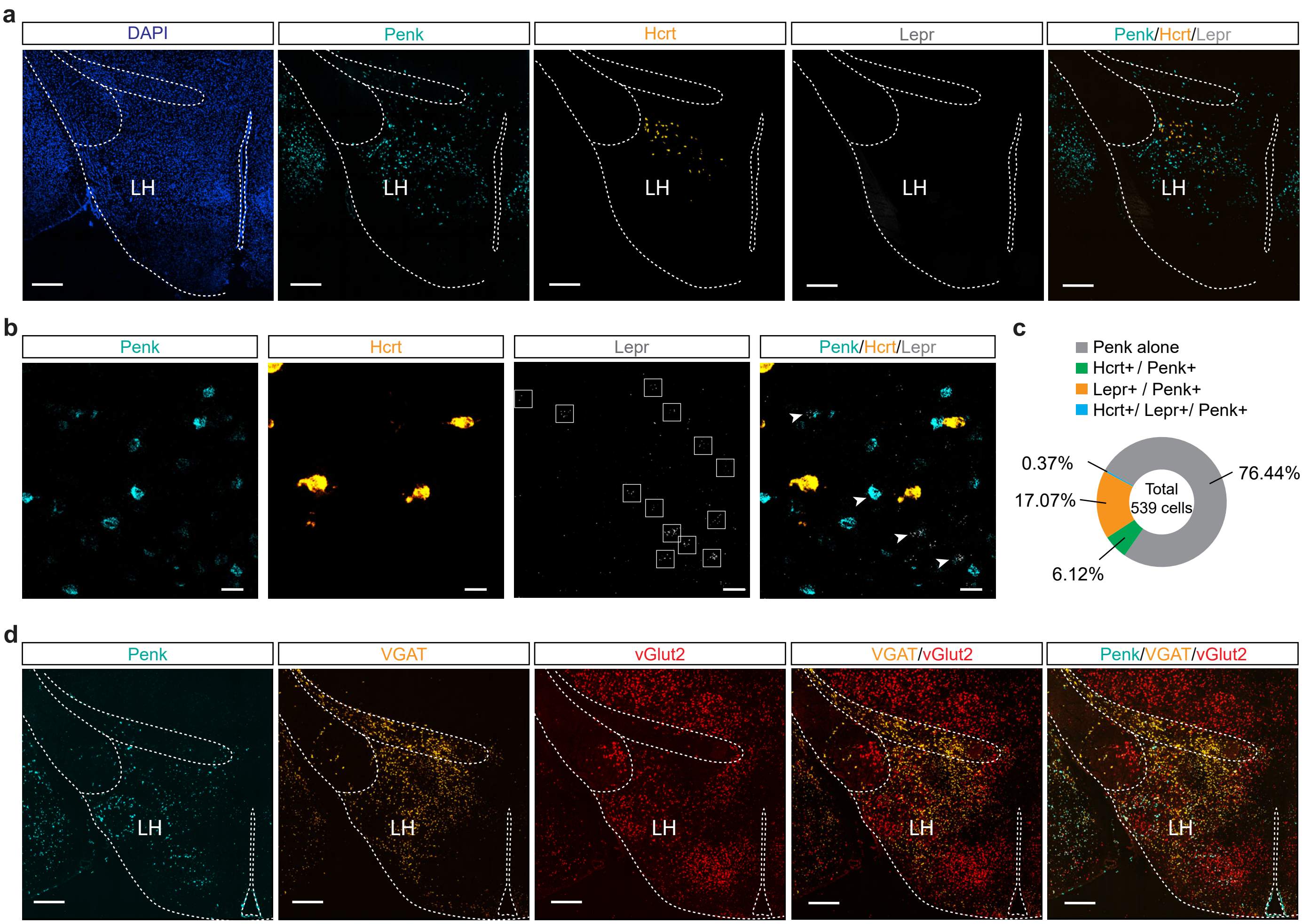

Supplementary Fig. 2

Supplementary Fig. 2 | Expression of Penk with other cell-type markers in the LH.

(a) Representative images of *in situ* hybridization for Penk, Hcrt, and Lepr in the LH. Scale bars, 250 µm.

(b) Enlarged views of a subregion of LH neurons expressing Penk, Hcrt, and Lepr in **a**; boxes show Lepr-expressing cells. Scale bars, 25 µm. Arrowheads represent colocalization between Penk and the other cell-type markers.

(c) Pie chart indicates the percentage of LH^{Penk} neurons colocalizing with Hcrt, Lepr, all, or none (n = 539 cells from two mice).

(d) Representative images of *in situ* hybridization for Penk, VGAT, and vGlut2 in the LH. Scale bars, 250 µm, replicated independently with similar results in three mice.

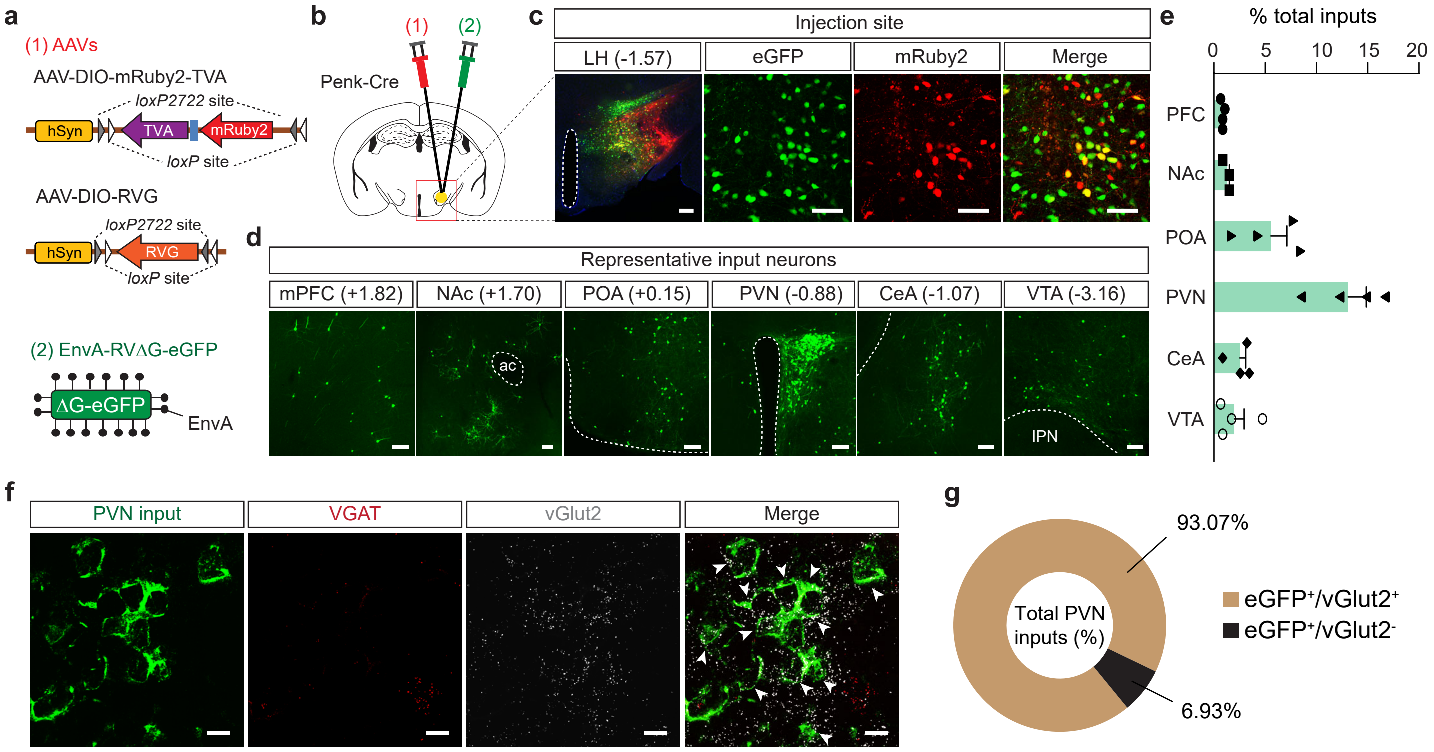

Supplementary Fig. 3

Supplementary Fig. 3 | Afferent connections of LH^{Penk} neurons.

(a, b) Injection regiment to map pseudotype rabies-mediated monosynaptic inputs to the LH^{Penk} neurons. AAV-DIO-mRuby2-TVA, AAV-DIO-RVG, and EnvA-RVΔG-eGFP were injected into the LH of Penk-Cre mice.

(c) The confocal image showing starter cells (yellow, expressing both eGFP and mRuby2) in the LH of Penk-Cre mice (left; scale bar, 200 μm), and its enlarged views showing Green, expressing eGFP; Red, expressing mRuby2; Yellow, expressing both eGFP and mRuby2. Scale bar, 50 μm.

(d) Representative images showing rabies-labeled presynaptic neurons in the mPFC, NAc, POA, PVN, CeA, and VTA. Scale bars, 100 μm.

(e) Quantitation of inputs to LH^{Penk} neurons showing the percentage of total cells in a given brain area relative to the total number of brain-wide inputs, replicated independently with three to four mice.

(f) Representative images of PVN neurons sending inputs to LH^{Penk} neurons with mRNA labeling for VGAT and vGlut2. Arrowheads represent colocalization of eGFP-expressing PVN neurons with vGlut2. Scale bars, 10 μm.

(g) Pie chart indicates the percentage of LH^{Penk}-projecting PVN neurons colocalizing with the probe to vGlut2 or not (n = 202 cells from two mice).

Data are presented as mean ± SEM.

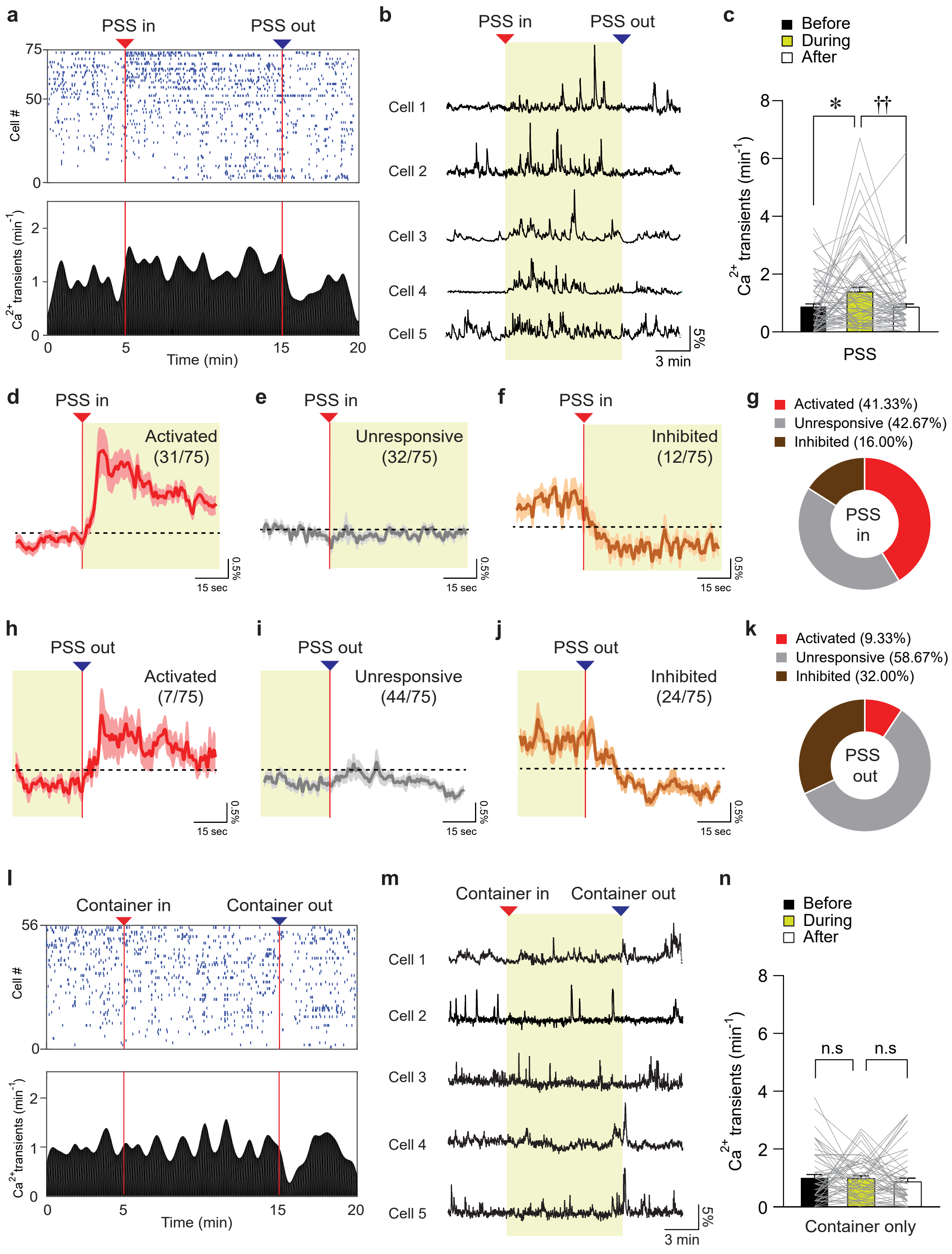

Supplementary Fig. 4

Supplementary Fig. 4 | *In vivo* imaging of Ca^{2+} dynamics of LH^{Penk} neurons in the presence of PSS.

(a) Raster plots (top) and peristimulus time histograms (bottom) showing LH^{Penk} neuronal activity of Penk-Cre mice in the presence of PSS (n = 75 cells from six Penk-Cre mice). The rows and ticks in the raster plots represent individual cells and single Ca^{2+} transient events, respectively. Vertical red bars mark the time the PSS was introduced (red arrow) or removed (blue arrow).

(b) Example traces of LH^{Penk} neuronal activity from five representative cells during 10-min PSS exposure. Light yellow shaded area indicates the presence of PSS.

(c) Average Ca^{2+} transients per min in LH^{Penk} neurons of Penk-Cre mice before, during, and after the presentation of PSS (n = 75 cells from six Penk-Cre mice). One-way RM ANOVA ($F_{(2,148)}$= 6.446, p = 0.002) was followed by Fisher LSD post hoc test for multiple comparisons; *p = 0.011 compared with before PSS; ††p = 0.001 compared with after PSS.

(d-g) Average trace of GCaMP6 fluorescence signal from activated (**d**), unresponsive (**e**), and inhibited (**f**) LH^{Penk} neurons in response to PSS introduction. Quantification of the % LH^{Penk} neurons showing activation, no-response, and inhibition upon PSS introduction (**g**).

(h-k) As for (**d-g**), but in response to PSS removal.

(l-n) As for (**a-c**), but in response to an empty container (n = 56 cells from five Penk-Cre mice). In **n**, one-way RM ANOVA ($F_{(2,110)}$= 0.443, p = 0.643) was followed by Fisher LSD post hoc test for multiple comparisons.

n.s., not significant. Data are presented as mean ± SEM.

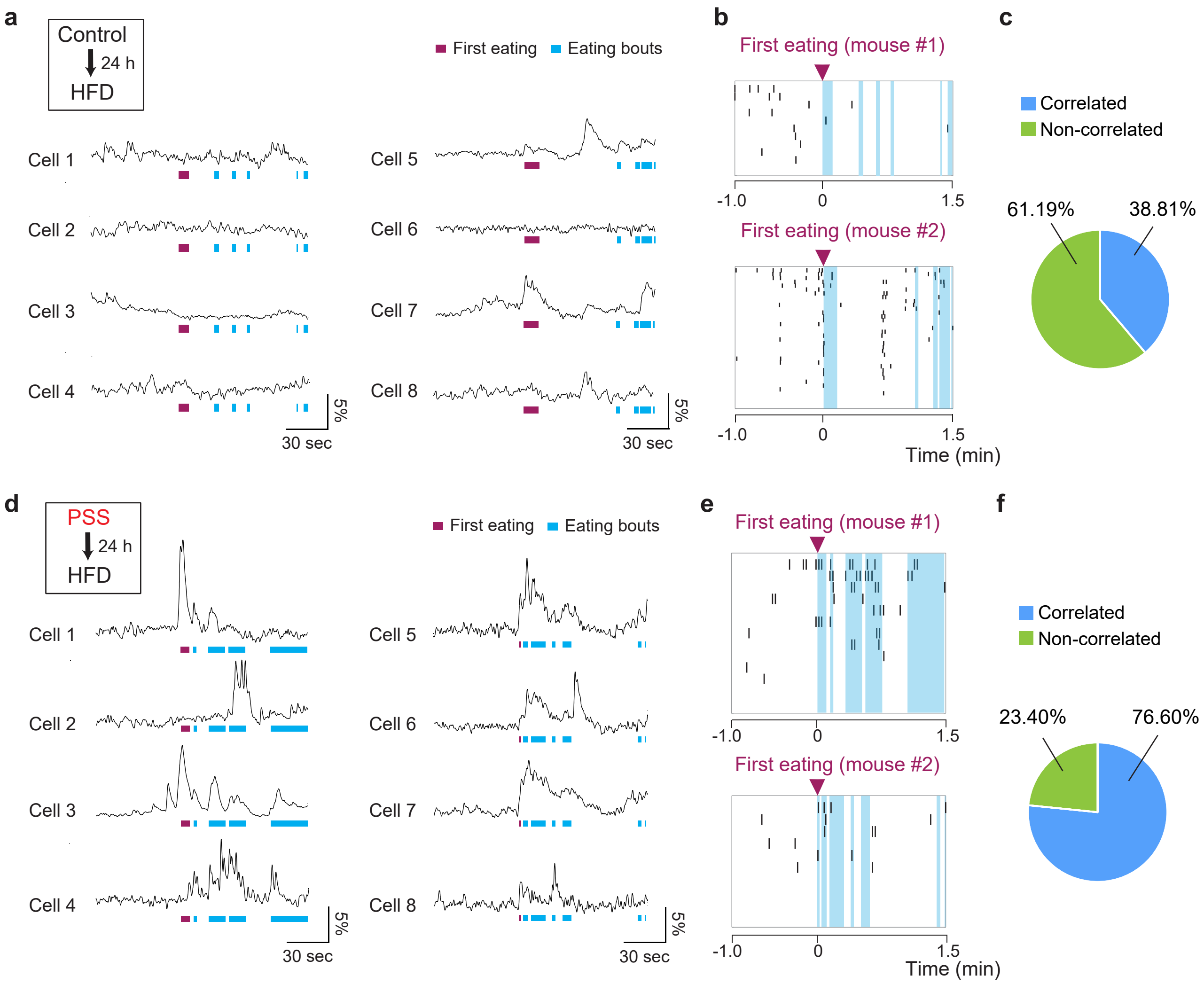

Supplementary Fig. 5

Supplementary Fig. 5 | *In vivo* activity of LH^{Penk} neurons during HFD consumption.

(a) Example traces of LH^{Penk} neuronal activity from eight representative LH^{Penk} neurons of two control Penk-Cre mice during HFD consumption. Magenta and light blue dashed areas indicate the first and following eating bouts, respectively.

(b) Raster plots showing LH^{Penk} neuronal activity from two representative control mice aligned to the first eating bout onset during HFD exposure. The rows and ticks in the raster plots represent individual cells and a single Ca^{2+} transient event, respectively. Light blue dashed areas indicate eating bouts.

(c) Pie charts indicate the % of Ca^{2+} transient events of control mice correlated with eating bout onset during the initial 1.5 min-recording after the first eating bout (n = 67 Ca^{2+} transients from four control Penk-Cre mice).

(d-f) As for (**a-c**), but for PSS mice during HFD consumption 24 h after PSS exposure; (n = 141 Ca^{2+} transients from five PSS Penk-Cre mice).

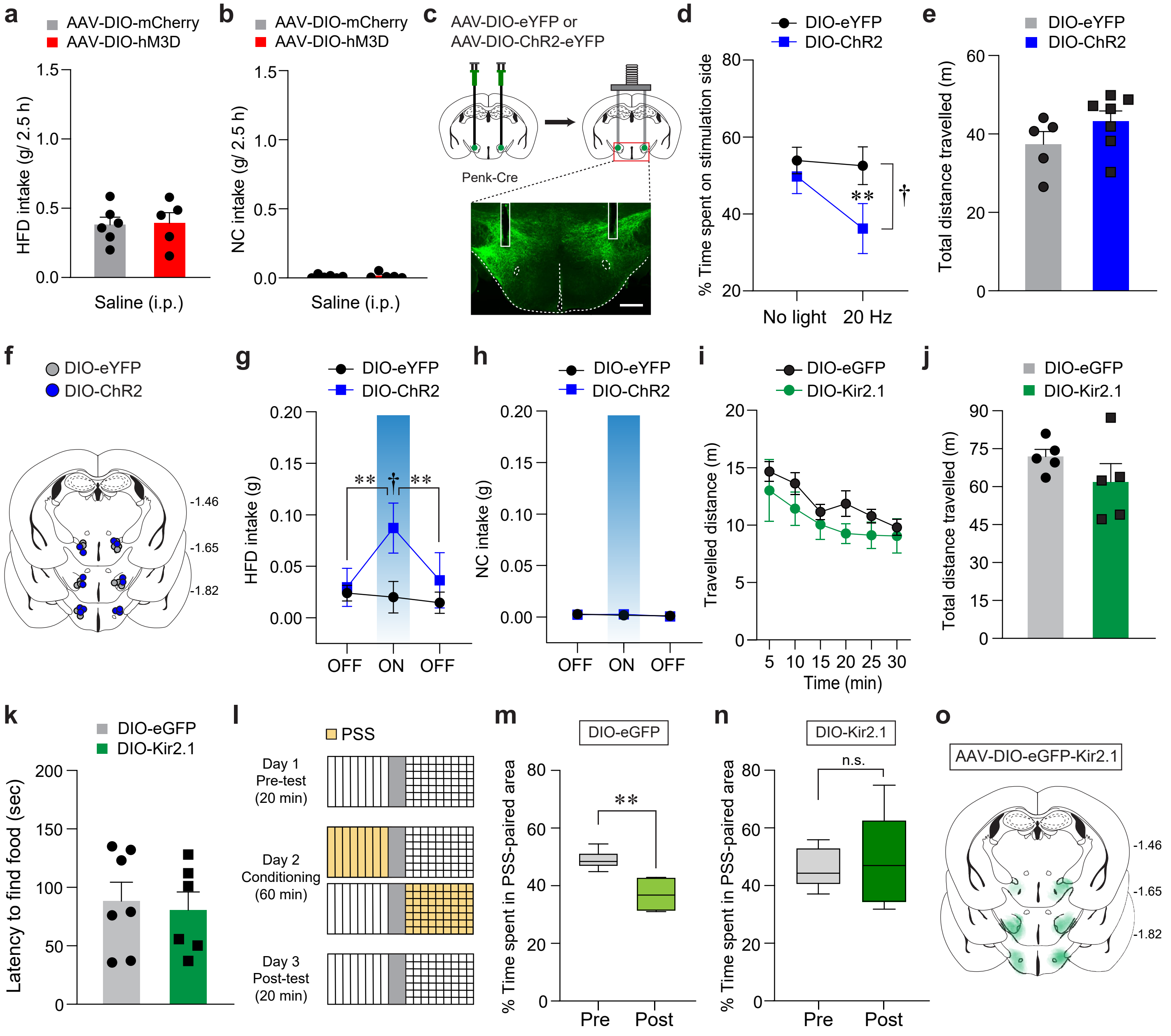

Supplementary Fig. 6

Supplementary Fig. 6 | Modulation of LH^{Penk} neuronal activity affects PSS-induced behavioral changes.

(a, b) 2.5 h HFD or NC consumption of saline-injected mice expressing either control virus (mCherry) or Gq-coupled DREADD (hM3D) in LH^{Penk} neurons (n = 6, 5 mice per group).

(c) Schematic depicting the injection of AAV-DIO-ChR2-eYFP, followed by implantation of optic fibers above the virus injection site in the LH of Penk-Cre (top). Representative images showing ChR2-eYFP expression in the LH^{Penk} neuronal cell bodies (bottom), replicated independently with similar results in seven mice. Scale bar, 250 μm.

(d) ChR2-expressing Penk-Cre mice spent less time in the photostimulated side of the RTPT chamber (n =7 mice per group). Two-way RM ANOVA ($F_{(1,12)}$= 5.598, p = 0.036) was followed by Bonferroni post hoc test for multiple comparisons; **p = 0.003 for no light vs. 20 Hz in ChR2-expressing mice; †p = 0.033 for eYFP- vs. ChR2-expressing mice at 20 Hz.

(e) Total distance travelled during the 15 min locomotion test in open field arena with photostimulation (n =5, 7 mice per group).

(f) Locations of the optic fibers included in **(d)**. Symbols represent the different groups: grey circle, AAV-DIO-eYFP; blue circle, AAV-DIO-ChR2-eYFP.

(g, h) Photostimulation of LH^{Penk} neurons increased HFD intake (**g;** n = 5 mice per group) but has no effect on NC consumption (**h**; n = 5 mice per group). In **g**, two-way RM ANOVA ($F_{(2,16)}$= 5.009, p = 0.020) was followed by Bonferroni post hoc test for multiple comparisons; **p = 0.003, **p = 0.008 for ChR2-expressing mice at pre-photostimulation vs. Light-ON and post-photostimulation vs. Light-ON, respectively; †p = 0.035 for control vs. ChR2-expressing mice at Light-ON.

(i, j) Locomotor activity analyzed in 5 min bins (**i**) and total distance travelled (**j**) during the 30 min test period (n = 5 mice per group).

(k) No significant difference in latency to find buried food was observed (n = 7, 6 mice per group).

(l) Experimental procedure to test CPA: one side of a two-sided chamber is paired with PSS on day 2.

(m, n) Percentage of time spent in PSS-paired side of mice expressing DIO-eGFP or DIO-Kir2.1 during

pre- and post-test (n = 6 mice per group). Box–whisker plots display median (center) and 2.5 to 97.5 percentiles of the distribution (bounds) with whiskers extending from min to max values. In **(m)**, two-tailed paired *t*-test, t_5= 4.072, **p = 0.00962. In **(n)**, two-tailed paired *t*-test, t_5= -0.524, p = 0.623.

(o) Summary diagram showing the coverage of eGFP-Kir2.1 viral infusion in the LH^{Penk} neurons of Penk-Cre mice in Fig. 4i, j.

n.s., not significant. Data are expressed as mean ± SEM.

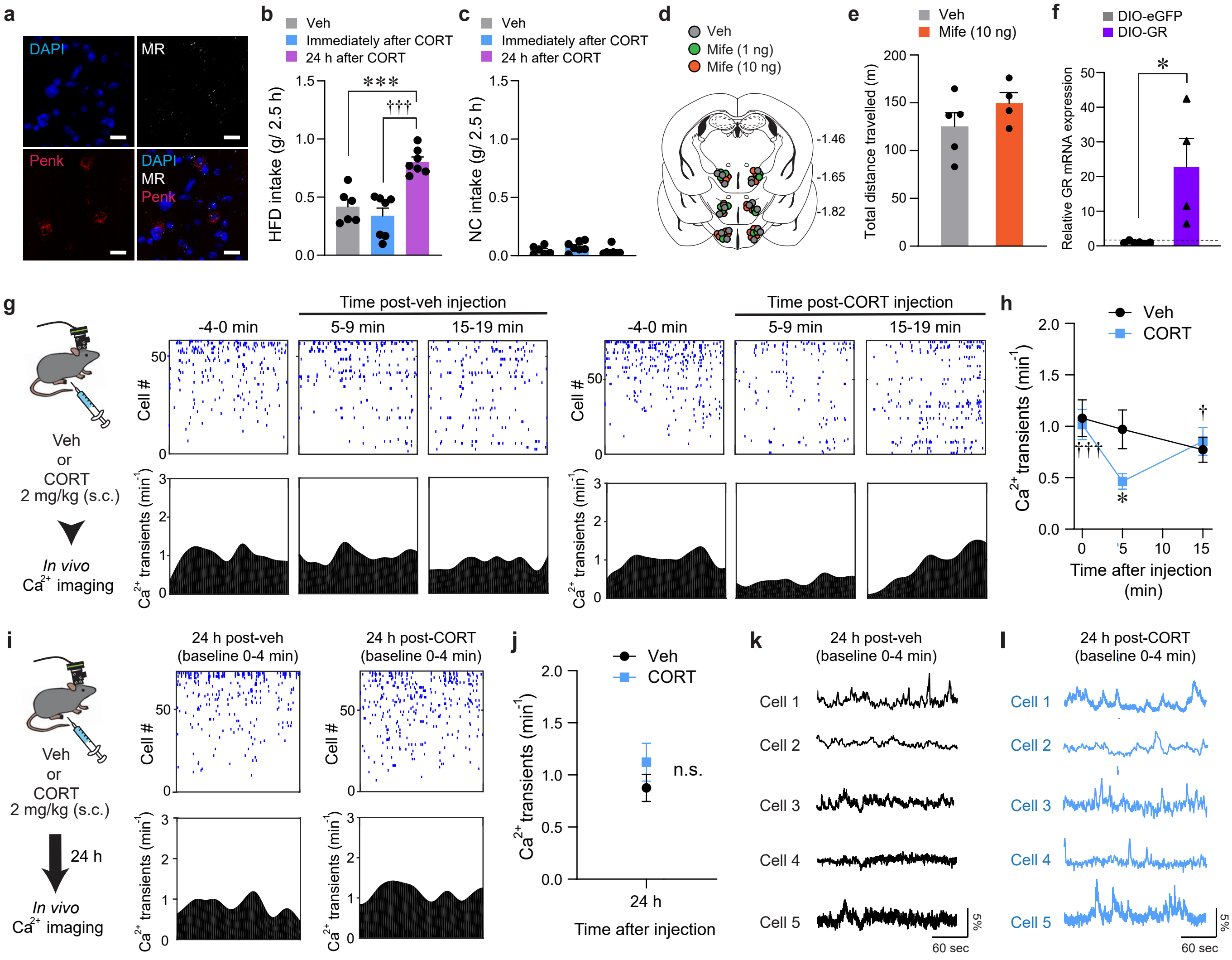

Supplementary Fig. 7

Supplementary Fig. 7 | CORT administration changes *in vivo* LH^{Penk} neuronal activity.

(a) Representative images of FISH for MR and Penk in the LH, replicated independently with similar results in three mice. Scale bars, 20 μm.

(b, c) 2.5 h HFD and NC consumption either immediately or 24 h after vehicle (veh) or CORT injection (2 mg/kg, s.c) (n = 6, 7, and 7 mice per group). In **(b),** one-way ANOVA ($F_{(2,17)}$= 19.559, $p < 0.001$) was followed by Fisher LSD post hoc test for multiple comparisons; ***$p < 0.001$, $^{†††}p < 0.001$ compared with HFD intakes 24 h after CORT injection (2 mg/kg, s.c.).

(d) Locations of the injection cannula tips in the mice included in Fig. 7g, h.

(e) The effects of local infusion of mifepristone (Mife; 10 ng/side) into the LH on locomotor activity (n = 5, 4 mice per group).

(f) qPCR analysis of GR mRNA expression in the LH (n = 6, 4 mice per group). Two-tailed unpaired *t*-test, t_8 = -3.282, *$p = 0.0111$.

(g) Raster plots and peristimulus time histograms showing LH^{Penk} neuronal activity of Penk-Cre mice after administration of veh (n = 57 cells from six mice) or CORT (n = 75 cells from seven mice). The rows and ticks in the raster plots represent individual cells and single Ca^{2+} transient events, respectively.

(h) Time course of changes in average Ca^{2+} transients per minute is measured during a 4-min window of pre-injection (at -4 min) and post-injection (at 5, 15 min) of veh (n = 57 cells from six mice) or CORT (n = 75 cells from seven mice). Two-way RM ANOVA ($F_{(2,260)}$= 3.805, $p = 0.024$) was followed by Bonferroni post hoc test for multiple comparisons; *$p = 0.014$ for veh- vs. CORT-treated mice at 5 min post-injection; $^{†††}p < 0.001$ for CORT-treated mice at baseline vs. 5 min post-injection; $^{†}p = 0.024$ for CORT-treated mice at 5 min vs. 15 min post-injection.

(i) Raster plots and peristimulus time histograms showing LH^{Penk} neuronal activity of Penk-Cre mice 24 h after administration of veh (n = 72 cells from seven mice) or CORT (n = 73 cells from seven mice). The rows and ticks in the raster plots represent individual cells and single Ca^{2+} transient events, respectively.

(j) Average Ca^{2+} transients per min in LH^{Penk} neurons of Penk-Cre mice during a 4-min baseline 24 h after administration of veh (n = 72 cells from seven mice) or CORT (n = 73 cells from seven mice). Two-tailed

unpaired *t*-test, t_{143}= -1.108, p = 0.270.

(k, l) Representative Ca^{2+} activity traces from LH^{Penk} neurons of Penk-Cre mice during a 4-min baseline 24 h after administration of veh (**k**) or CORT (**l**).

n.s., not significant. Data are expressed as mean ± SEM.

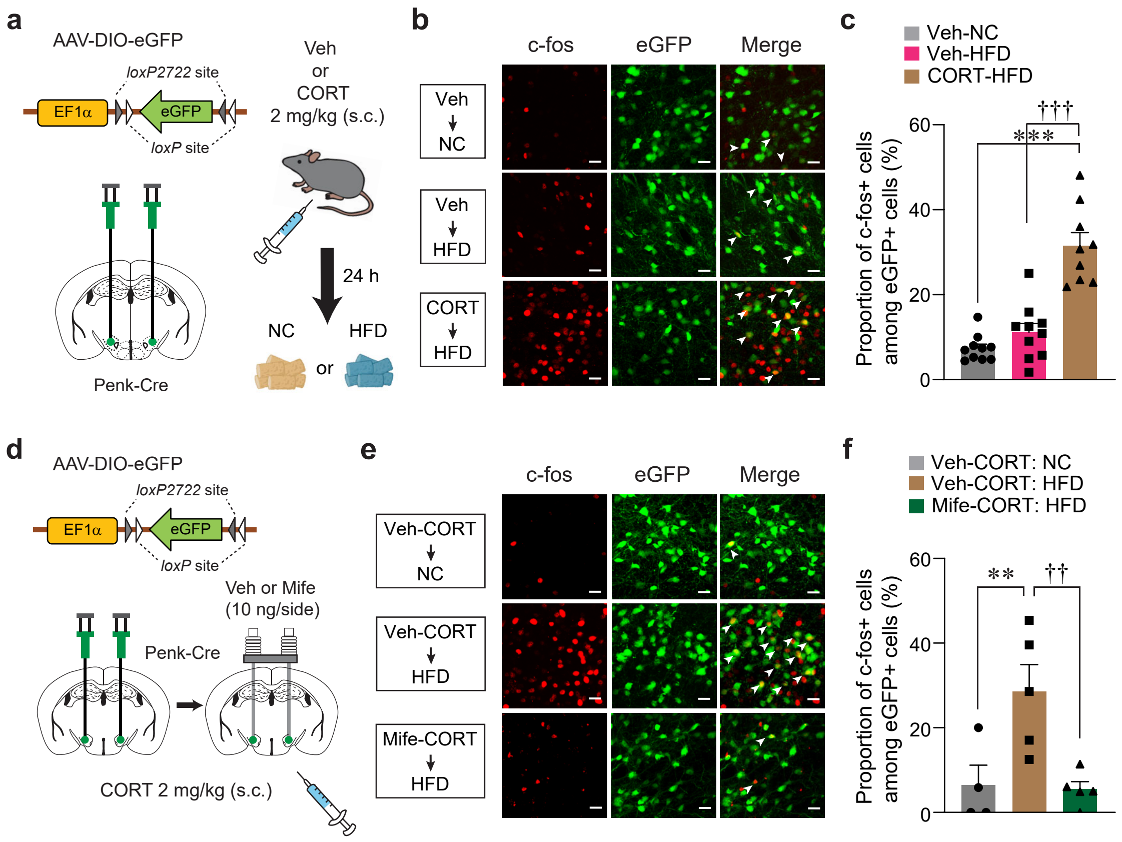

Supplementary Fig. 8

Supplementary Fig. 8 | CORT pretreatment changes HFD-induced c-fos expression in LH^{Penk} neurons. (a) Schematic for the bilateral injection of AAV-DIO-eGFP in the LH of Penk-Cre mice (left). HFD is presented 24 h after veh or CORT injection (right).

(b) Representative images showing c-fos immunoreactivity (red) and eGFP fluorescence (green) in the LH of veh- or CORT-treated mice in response to NC or HFD exposure at 24 h-post injection. Arrowheads indicate the colocalization of c-fos with Penk expression. Scale bars, 25 μm.

(c) Quantification of the proportion of c-fos-positive cells among LH^{Penk} neurons in **b** (n = 10, 10, and 9 mice per group). One-way ANOVA ($F_{(2,26)}$= 36.086, $p < 0.001$) was followed by Fisher LSD post hoc test for multiple comparisons; ***$p < 0.001$ compared with veh-treated mice exposed to NC only; $^{†††}p < 0.001$ compared with veh-treated mice exposed to HFD.

(d) Schematic for the bilateral injection of AAV-DIO-eGFP into the LH of Penk-Cre mice followed by cannula implantation into the same site for microinfusion of veh or mifepristone (Mife). Systemic CORT injection was subsequently performed after the microinfusion.

(e) Representative images showing c-fos immunoreactivity (red) and eGFP fluorescence (green) in the LH in response to either NC or HFD 24 h after microinfusion of veh or Mife and CORT injection (2 mg/kg, s.c.). Arrowheads indicate the colocalization of c-fos with Penk expression. Scale bars, 25 μm.

(f) Quantification of the proportion of c-fos-positive cells among LH^{Penk} neurons in **e** (n = 4, 5, and 5 mice per group). One-way ANOVA ($F_{(2,11)}$= 8.093, $p = 0.007$) was followed by Fisher LSD post hoc test for multiple comparisons; **$p = 0.008$, $^{††}p = 0.004$ compared with mice exposed to HFD 24 h after microinfusion of veh and CORT injection.
