# Supplementary material for: Lateral hypothalamic proenkephalin neurons drive threat-induced overeating associated with a negative emotional state
Source: Nat Commun. 2023 Oct 28;14:6875. doi: 10.1038/s41467-023-42623-6 (PMC10613253; doi:10.1038/s41467-023-42623-6)
Supplement: Supplementary file 3 — Reporting Summary [file 41467_2023_42623_MOESM3_ESM.pdf]

nature portfolio

Corresponding author(s): Sora Shin

Last updated by author(s): Sept/3/2023

Reporting Summary

Nature Portfolio wishes to improve the reproducibility of the work that we publish. This form provides structure for consistency and transparency in reporting. For further information on Nature Portfolio policies, see our Editorial Policies and the Editorial Policy Checklist.

Statistics

For all statistical analyses, confirm that the following items are present in the figure legend, table legend, main text, or Methods section.

n/a	Confirmed	
☐	☒	The exact sample size (*n*) for each experimental group/condition, given as a discrete number and unit of measurement
☐	☒	A statement on whether measurements were taken from distinct samples or whether the same sample was measured repeatedly
☐	☒	The statistical test(s) used AND whether they are one- or two-sided<br>*Only common tests should be described solely by name; describe more complex techniques in the Methods section.*
☒	☐	A description of all covariates tested
☐	☒	A description of any assumptions or corrections, such as tests of normality and adjustment for multiple comparisons
☐	☒	A full description of the statistical parameters including central tendency (e.g. means) or other basic estimates (e.g. regression coefficient) AND variation (e.g. standard deviation) or associated estimates of uncertainty (e.g. confidence intervals)
☐	☒	For null hypothesis testing, the test statistic (e.g. *F*, *t*, *r*) with confidence intervals, effect sizes, degrees of freedom and *P* value noted<br>*Give P values as exact values whenever suitable.*
☒	☐	For Bayesian analysis, information on the choice of priors and Markov chain Monte Carlo settings
☒	☐	For hierarchical and complex designs, identification of the appropriate level for tests and full reporting of outcomes
☒	☐	Estimates of effect sizes (e.g. Cohen's *d*, Pearson's *r*), indicating how they were calculated

Our web collection on statistics for biologists contains articles on many of the points above.

Software and code

Policy information about availability of computer code

Data collection	Inscopix nVoke 2.0; Inscopix Data acquisition software (IDAS, v 1.6.1); ANY-maze (Stoelting Co); Zen 2012 Sp5 imaging software (Carl Zeiss); NIS-Elements imaging software (v 5.21.03, Nikon Microscope)
Data analysis	Inscopix Data processing software (IDPS, v 1.6.1); MATLAB R2015a (Mathworks); FIJI (v2.5, ImageJ, NIH); GraphPad Prism 9; SigmaStat (v 4.0, Inpixon)

For manuscripts utilizing custom algorithms or software that are central to the research but not yet described in published literature, software must be made available to editors and reviewers. We strongly encourage code deposition in a community repository (e.g. GitHub). See the Nature Portfolio guidelines for submitting code & software for further information.

Data

Policy information about availability of data

All manuscripts must include a data availability statement. This statement should provide the following information, where applicable:

- Accession codes, unique identifiers, or web links for publicly available datasets
- A description of any restrictions on data availability
- For clinical datasets or third party data, please ensure that the statement adheres to our policy

The data that support the findings of this study are available from the corresponding author upon reasonable request because of the size and complexity of the datasets.

Research involving human participants, their data, or biological material

Policy information about studies with human participants or human data. See also policy information about sex, gender (identity/presentation), and sexual orientation and race, ethnicity and racism.

Reporting on sex and gender	N/A
Reporting on race, ethnicity, or other socially relevant groupings	N/A
Population characteristics	N/A
Recruitment	N/A
Ethics oversight	N/A

Note that full information on the approval of the study protocol must also be provided in the manuscript.

Field-specific reporting

Please select the one below that is the best fit for your research. If you are not sure, read the appropriate sections before making your selection.

[x] Life sciences [ ] Behavioural & social sciences [ ] Ecological, evolutionary & environmental sciences

For a reference copy of the document with all sections, see nature.com/documents/nr-reporting-summary-flat.pdf

Life sciences study design

All studies must disclose on these points even when the disclosure is negative.

Sample size	Samples size for each experiment is described in the figure legend. Initially, sample sizes required for this study were estimated based on pilot studies or previous work (e.g., Shin et al., 2022, Nature Neuroscience; Knowland et al., 2017, Cell). Power analysis was also conducted to validate the sample size and the endpoint, according to Sample Size Determination (significance level at 0.05 and power at 0.9) from the NIH "Guidelines for the Care and Use of Mammals in Neuroscience and Behavioral Research".
Data exclusions	If the cannula tips were found outside reference area of the LH or the viral transduction was weak (covering less than 50% of the total LH area), we excluded the mice from the final dataset, which was determined by two experimenters who were blinded to the experimental design. This exclusion happens in one C57BL6/J mouse in Fig 7f due to off-target cannula implantation; one Penk-Cre mouse in each Fig. 4i and Fig. 6d due to weak viral transduction.
Replication	Results were replicated in multiple trials within each animal and/or across different animals within each data set. Reproduction of the data was considered successful if the same trends were observed in the multiple trials. Experiments were replicated several rounds until we meet the criteria of power analysis. The experiments were performed independently two to three times for in situ hybridization; three to four times for viral tracing study; four to five times for in vivo calcium imaging; minimum five times for behavioral tests.
Randomization	Male and female mice aged 12-20 weeks were randomly picked and used. Within a group with or without PSS history, we randomly chose animals for experiments. Animals used in this study were not selected based on any other prerequisite features other than general animal wellbeing (e.g. normal grooming and social behavior, no obvious infections, etc.) for allocation into a particular experimental group.
Blinding	Experimenters are blind to the group allocation and outcome assessment. Data analysis was carried out without the subjective bias. The video analysis in Fig. 3e-r; Fig. 7l-n; Extended Data Fig. 5; Extended Data Fig 6k was performed by two experimenters who were blinded to the experimental design.

Reporting for specific materials, systems and methods

We require information from authors about some types of materials, experimental systems and methods used in many studies. Here, indicate whether each material, system or method listed is relevant to your study. If you are not sure if a list item applies to your research, read the appropriate section before selecting a response.

Materials & experimental systems

n/a	Involved in the study	
☐	☒	Antibodies
☐	☒	Eukaryotic cell lines
☒	☐	Palaeontology and archaeology
☐	☒	Animals and other organisms
☒	☐	Clinical data
☒	☐	Dual use research of concern
☒	☐	Plants

Methods

n/a	Involved in the study	
☒	☐	ChIP-seq
☒	☐	Flow cytometry
☒	☐	MRI-based neuroimaging

Antibodies

Antibodies used	anti-c-fos (Cell Signaling Technology; Cat# 2250S); Goat anti-Rabbit IgG (H+L) Highly Cross-Adsorbed Secondary Antibody, Alexa Fluor Plus 555 (Thermo Fisher Scientific; Cat# A32732; Lot# XI353661)
Validation	Anti-c-fos (Cell Signaling Technology; Cat# 2250S, RRID:AB_2247211): Validated in previous studies with many citations (Ref. Shin et al., 2018, Neuron; Shin et al., 2022, Nat. Neurosci; Pribiag et al., 2021, Neuron). Information can be found in this website. https://www.cellsignal.com/products/primary-antibodies/c-fos-9f6-rabbit-mab/2250<br><br>Goat anti-Rabbit IgG (H+L) Highly Cross-Adsorbed Secondary Antibody, Alexa Fluor Plus 555 (Thermo Fisher Scientific; Cat# A32732, RRID:AB_2633281): Validated in previous studies with many citations. Information can be found in this website. https://www.thermofisher.com/antibody/product/Goat-anti-Rabbit-IgG-H-L-Highly-Cross-Adsorbed-Secondary-Antibody-Polyclonal/A32732

Eukaryotic cell lines

Policy information about cell lines and Sex and Gender in Research

Cell line source(s)	HEK293 cell is purchased from Agilent Technologies (https://www.agilent.com/en/product/protein-expression/proteinexpression-vectors-kits/viral-mediated-delivery-systems/aav-293-cells-232993)
Authentication	No authentication was performed on this cell line.
Mycoplasma contamination	No contamination was reported during the experimental period. The cells were identified as mycoplasma negative.
Commonly misidentified lines (See ICLAC register)	No commonly misidentified cell lines were used in this study.

Animals and other research organisms

Policy information about studies involving animals; ARRIVE guidelines recommended for reporting animal research, and Sex and Gender in Research

Laboratory animals	12-20-week-old adult C57BL6/J mice, Penk-Cre mice (Stock No. 025112), Lepr-Cre mice (Stock No. 008320) and Ai14 mice (Stock No. 007908; tdTomato reporter line) were attained from Jackson Laboratories. All transgenic mice were backcrossed to wild-type C57BL/6J mice for multiple generations. Mice were group-housed, given access to food pellets and ad libitum, and maintained on a 12 hr:12 hr light:dark cycle (lights on at 7:00 AM) with standard bedding in a temperature- and humidity-controlled room (~21°C and 42% humidity).
Wild animals	This study did not involve wild animals.
Reporting on sex	Both male and female animals were used.
Field-collected samples	This study did not involve samples collected from the field.
Ethics oversight	All experiments were carried out in accordance with US National Institutes of Health guidelines and approved by the Virginia Polytechnic Institute and State University institutional animal care and use committee (IACUC).

Note that full information on the approval of the study protocol must also be provided in the manuscript.
